# The importance of publishing research protocols for pharmacoeconomic studies

**DOI:** 10.1136/ejhpharm-2021-002987

**Published:** 2021-09-15

**Authors:** Aniek Dane, Anne-Sophie Klein Gebbink, P Hugo M van der Kuy

**Affiliations:** Department of Pharmacy, Erasmus Medical Center, Rotterdam, The Netherlands

**Keywords:** economics, pharmaceutical, commerce, pharmacy administration, research design, clinical trial

Bearing in mind that it is currently not standard practice to publish non-clinical trial research protocols in *EJHP*, we would like to share our thoughts within the *EJHP* community on considering publishing these kinds of research protocols. We believe drawing attention to both the academic and societal relevance of submitting non-clinical trial research protocols, especially in the field of pharmacoeconomics, may foster discourse on this delicate subject. To reinforce our arguments we have attached our research protocol for the study ‘Orphan Drug Prices and Market Exclusivity in Western-European Countries’ (see online supplemental file 1).

10.1136/ejhpharm-2021-002987.supp1 Supplementary data

Primarily, publishing a research protocol is a means to allow the academic community to evaluate whether subsequent analysis and results are in line with the investigators’ initial objectives. Additionally, it informs the academic community on ongoing research and may avoid duplication of work.

It can be argued that providing such insight is especially relevant for clinical trials from a methodological or procedural point of view. Nevertheless, this holds the same for pharmacoeconomic studies. Drawing robust conclusions from these kind of studies is highly dependent on applied research techniques. Publishing pharmacoeconomic research protocols can contribute to the overall quality of research techniques and subsequent credibility of conclusions, because it enhances transparency and accountability.

To support this argument and to illustrate the particular relevance of publishing the research protocol for our study on orphan drug prices, we list our main justifications below:

- Our study collects data on actual purchasing (post-discount) prices of orphan drugs, which are subject to substantial confidentiality constraints. This calls for a high degree of justification of methodologies to demonstrate integrity and confirmation to confidentiality challenges. Publication of a research protocol can be a means to ensure transparency on these matters. Subsequently, it can provide insight into potential ways of dealing with such confidentiality challenges for various scientific and societal stakeholders.
- Criticism following publications on drug (list) prices may involve accusations of ‘cherry picking’. This involves stakeholders arguing that, instead of including drugs for price analysis in an objective manner, drugs included in such analyses are known in advance to have remarkable prices. Hence, for the societal impact and credibility of our study results, it is relevant to provide transparency on the inclusion criteria applied upfront to potentially avoid devaluation of valuable results.
- Our study protocol addresses a highly underexposed research area with a great need for data to evaluate policy and inform future policy making on orphan drug pricing. It is highly challenging to collect actual purchasing prices of drugs, let alone orphan drugs, and no similar studies have been published to date, to the best of our knowledge. Therefore, publication of our research protocol is potentially of high value to academic and other stakeholders as it offers transparency on applied study procedures and potentially fosters future research.

For the reasons stated above and potentially to nourish discourse on the relevance of publishing this protocol as well as similar types of research protocols in the future, we invite *EJHP* stakeholders to reflect on this letter.

