# Supplementary material for: The importance of publishing research protocols for pharmacoeconomic studies
Source: Eur J Hosp Pharm. 2021 Sep 15;30(1):e4. doi: 10.1136/ejhpharm-2021-002987 (PMC9811535; doi:10.1136/ejhpharm-2021-002987)
Supplement: Supplementary data [file ejhpharm-2021-002987supp001.pdf]

Title

Research protocol: Orphan drug prices and market exclusivity in Western-European countries: a multi-institutional, mixed-method study

Abstract

Introduction

High prices for Orphan Medicinal Products (OMPs) have become a controversial issue for various stakeholders and research has raised questions about OMPs' value and appropriate price levels. One of the main challenges is to put in place effective incentives to foster innovation while ensuring justifiable prices. However, due to confidentiality issues transparency on purchasing prices of OMPs is lacking. Therefore, research is needed to gain insight in purchasing prices to support policy making. The aim of this study is to analyse price developments of OMPs after market exclusivity expiry and to explore their subsequent market mechanisms.

Methods and analysis

Both list prices and purchasing prices from a preselected group of OMPs will be collected from university hospitals in a number of Western-European countries. Further, semi-structured interviews will be conducted with the Heads of Pharmacy and drug purchasers employed at the participating hospitals.

Ethics and dissemination

Price data will be obtained through the data capture tool Castor EDC and stored at a secured saver at Erasmus Medical Center, Rotterdam, the Netherlands. All data will be subject to pseudonymization to confirm to confidentiality restrictions. Results may inform decision-makers and other stakeholders and potentially contribute to finding adequate policy solutions.

Introduction

High drug prices have been subject to debate at policy tables across Europe since the 1990ies and have raised concerns about the ethics and sustainability of pharmaceutical pricing. High drug prices may result in displacement of other types of care because of limited healthcare budgets and may lead to reduced accessibility for patients who need medicines.[1–3]

Especially pricing of drugs for rare diseases, Orphan Medicinal Products (OMPs), has become a source of concern for payers and other stakeholders.[4–7] Previous research has raised questions about acceptable price levels, cost-benefit ratios, as well as value of orphan drugs.[8–10]

Due to small patient populations and subsequent low expected market rewards, it is challenging to incentivise research and development (R&D) for OMPs.[1,3] In line with this, relatively little progress is made in treatments for rare diseases.[11] The European Commission states that patients with rare diseases "should be entitled to the same quality of treatment as other patients" and adopted Regulation (EC) No 141/2000 in 2000. The Regulation attempts to incentivise R&D and market entry for OMPs for pharmaceutical companies.[12,13]

One of the key incentives set out in the Regulation is ten years of market exclusivity for new OMPs. During the period of market exclusivity, the market authorisation holder is assured that competitors are withhold from market entry within the EU.[13]

Although the Regulation seems to foster innovation, high prices of OMPs often remain intact after expiration of the market exclusivity period. As of today, one of the main challenges in regulating the OMP market is to apply effective incentives for innovation while ensuring justifiable prices.[10,14,15]

A lack of transparency from the industry-side on both R&D costs and pricing systems is considered a barrier in addressing the issue of pharmaceutical pricing.[3,16] Purchasing prices of drugs are rarely published due to sensitivity and confidentiality issues.[17] We found only one study that attempts to analyse actual drug prices instead of list prices. Van Harten et al (2016) compared actual prices of cancer drugs in a number of European countries. They underline that substantial differences exist between list prices and actual prices and between countries.[18] Nevertheless, to our knowledge, such analysis does not exist for OMPs specifically.

We need to gain insight in purchasing prices of OMPs to adequately assess underlying market mechanisms and to address the challenge of high pricing. A study offering such insight would support decision-makers in finding policy solutions that help containing costs without preventing patients' needs being met. Correspondingly, this study aims to a) analyse both list and purchasing price developments of OMPs in Western European countries; and b) to explore potential explanations for observed price developments.

Methods and analysis

A foundation for a multi-institutional, mixed-methods study is set out in which both quantitative and qualitative data will be collected on OMP pricing. The Heads of Pharmacy from a number of university hospitals across Western-Europe are invited to participate in the study and share their price data with the research team at Erasmus Medical Center, Rotterdam (Erasmus MC). Selection of hospitals and subsequent invitation to participate are based on professional networks of the authors. When a hospital is reluctant to participate in our study, we assess underlying reasons to address potential selection bias.

Since OMPs are predominantly prescribed at university hospitals, we aim to take on a university hospital purchasers' perspective.

Inclusion criteria

To draw general conclusions about the orphan drug market, we will select a group of diverse OMPs. First, we apply the following inclusion criteria: OMP has orphan designation and OMP has had market exclusivity that expired no later than 31 December 2019. Both criteria are based on records of European Commission decisions as set out by Regulation (EC) No 141/2000.[13] Moreover, OMPs that are withdrawn from the market are excluded as well as products without price data at Erasmus MC.

Second, we apply three criteria to create distinct OMP categories: prevalence of underlying indication (<5 per 10.000 persons for orphan drugs and <2 per 100.000 persons for ultra-orphan drugs), route of administration (enteral or parenteral) and therapeutic modality (small-molecule or biological). As the combination of biological and enteral administration does not exist, six categories are formed (Table 1).

Table 1 - OMP categories

UOD + S + E	UOD + S + P	UOD + B +P
OD + S + E	OD + S + P	OD + B + P

UOD: ultra-orphan drug OD: orphan drug
S: small molecule B: biological
E: enteral P: parenteral

Third, we identify four scenarios of drug competition that may be at hand after expiration of the market exclusivity period: 1) OMP can be considered generic itself; 2) OMP is subject to competition with generics; 3) OMP is subject to competition with other patented drugs; 4) no competition is observed. From each of the six categories, one OMP will be selected from each of the four competition scenarios. Notably, not every competition scenario is observed in each category of OMPs. Hence, an estimated 12-14 OMPs will be included.

Data collection

A template consisting of an overview of selected OMPs and their authorized representations will be sent to the participating university hospitals. Corresponding prices are requested in averages per year per primary sales unit to correct for different pack sizes. For example, the average price of '*Brand Drug X Capsule 25 mg'* per one capsule.

Administrative in-house records will be consulted to extract historical purchasing prices. List prices may be obtained through external parties such as government institutions or insurers. Prices will be adjusted for VAT-differences to enable cross-country comparison.

Further, semi-structured interviews will be held with purchasers at participating university hospitals to explore potential explanations for observed price trends, taking into account country-specific factors such as reimbursement frameworks.

Outcomes

OMP price trends and differences will be stated in proportions and clustered into group averages to avoid traceability to specific prices for purchasing hospitals in light of confidentiality constraints. Findings from the interviews will be summarized in the results section and used to critically assess our quantitative results in the discussion section of the research report.

Data analysis

To assess whether list prices and purchasing prices differ significantly, means testing will be performed using IBM SPSS Statistics. Price developments relative to market exclusivity will be analyzed using interrupted time series analysis. During the interviews, drug purchasers are asked about market mechanisms that may help to explain observed price trends. Data from the interviews will be coded and is subject to deductive analysis.

Moreover, with our analyses of price data we aim to identify and quantify trends whereas the interviews aim to explore causal connections.

Ethics and dissemination

No medical or patient data will be collected; hence this study does not fall under the scope of the Dutch Medical Research Involving Human Subjects Act. Data may contain personal information or information that can identify individuals so the General Data Protection Regulation (GDPR) is applicable. Price data collected from participating hospitals can be considered sensitive and is subject to confidentiality constraints. We aim to find ways to overcome this challenge in light of the scientific and societal relevance of our research. For instance, data will be pseudonymized and presented in group averages. Further, data will be surveyed through Castor EDC and saved at a secured server at Erasmus MC. This server is only accessible for members of the research team.

Acknowledgements
The authors wish to thank Jan-Dietert Brugma and Inger Snijders, both employed at the Department of Pharmacy Erasmus Medical Center Rotterdam, for their contributions to extract price data from the hospital pharmacy records and their expert views on establishing a transparent OMP selection strategy.

References

1. OECD/European Union. Health at a Glance: Europe 2020: State of Health in the EU Cycle. Chapter 5: Health Expenditure and Financing. OECD Publishing. 2020.
2. Vogler S, Paris V, Ferrario A, Wirtz VJ, de Joncheere K, Schneider P, et al. How Can Pricing and Reimbursement Policies Improve Affordable Access to Medicines? Lessons Learned from European Countries. Appl Health Econ Health Policy. 2017 Jun;15(3):307–21.
3. OECD. Pharmaceutical Innovation and Access to Medicines, OECD Health Policy Studies [Internet]. OECD Publishing. Paris; 2018. Available from: https://doi.org/10.1787/9789264307391-en
4. Szegedi M, Zelei T, Arickx F, Bucsics A, Cohn-Zanchetta E, Fürst J, et al. The European challenges of funding orphan medicinal products. Orphanet J Rare Dis. 2018 Nov;13(1):184.
5. Bourdoncle M, Juillard-Condat B, Taboulet F. Patient access to orphan drugs in France. Orphanet J Rare Dis. 2019 Feb;14(1):47.
6. Garattini L, Padula A. Pharmaceutical expenditure control in Europe: time to move from pricing to budgeting? J R Soc Med. 2020 Mar;113(3):93–7.
7. Michel M, Toumi M. Access to orphan drugs in Europe: current and future issues. Expert Rev Pharmacoecon Outcomes Res. 2012 Feb;12(1):23–9.
8. Schoser B, Hahn A, James E, Gupta D, Gitlin M, Prasad S. A Systematic Review of the Health Economics of Pompe Disease. PharmacoEconomics - open. 2019 Dec;3(4):479–93.
9. Berdud M, Drummond M, Towse A. Establishing a reasonable price for an orphan drug. Cost Eff Resour Alloc [Internet]. 2020;18(1):1–18. Available from: https://doi.org/10.1186/s12962-020-00223-x
10. Mestre-Ferrandiz J, Palaska C, Kelly T, Hutchings A, Parnaby A. An analysis of orphan medicine expenditure in Europe: is it sustainable? Orphanet J Rare Dis. 2019 Dec;14(1):287.
11. Giannuzzi V, Landi A, Bosone E, Giannuzzi F, Nicotri S, Torrent-Farnell J, et al. Failures to further developing orphan medicinal products after designation granted in Europe: an analysis of marketing authorisation failures and abandoned drugs. BMJ Open. 2017 Sep;7(9):e017358.
12. European Medicines Agency. Legal framework: orphan designation | European Medicines Agency [Internet]. [cited 2021 Mar 4]. Available from: https://www.ema.europa.eu/en/human-regulatory/overview/orphan-designation/legal-framework-orphan-designation
13. The European Parliament and the Council of the European Union. Regulation (EC) No 141/2000 of the European Parliament and of the Council of 16 December 1999 on orphan medicinal products. Off J Eur Union [Internet]. 2000;L 18:1–5. Available from: https://eur-lex.europa.eu/legal-content/EN/TXT/PDF/?uri=CELEX:32000R0141&from=EN
14. Gronde T van der, Uyl-de Groot CA, Pieters T. Addressing the challenge of high-priced prescription drugs in the era of precision medicine: A systematic review of drug life cycles, therapeutic drug markets and regulatory frameworks. PLoS One. 2017;12(8):e0182613.
15. European Commission. Joint evaluation of Regulation (EC) No 1901/2006 of the European Parliament and of the Council of 12 December 2006 on medicinal products for paediatric use and Regulation (EC) No 141/2000 of the European Parliament and of the Council of 16 December 1999 o [Internet]. 2020. Available from: https://ec.europa.eu/health/sites/health/files/files/paediatrics/docs/orphan-regulation_eval_swd_2020-163_part-1.pdf

16. Brouwer W, van Baal P, van Exel J, Versteegh M. When is it too expensive? Cost-effectiveness thresholds and health care decision-making. Eur J Heal Econ [Internet]. 2019;20(2):175–80. Available from: http://dx.doi.org/10.1007/s10198-018-1000-4
17. Block, de M. The difficulty of camparing drug prices between countries. Lancet Oncol. 2016;17:125.
18. van Harten WH, Wind A, de Paoli P, Saghatchian M, Oberst S. Actual costs of cancer drugs in 15 European countries. Lancet Oncol. 2016;17(1):18–20.

Authors' contributions
Aniek Dane: designed the study and was involved in planning and supervised the work.
Anne-Sophie Klein Gebbink: involved in designing the study and wrote the protocol with input of all the authors.
P. Hugo M. van der Kuy: supervises the project.
Carin A. Uyl-de Groot: involved in developing the theoretical framework. All authors provided critical feedback and gave approval for submission.

Funding statement
This research received no specific grant from any funding agency in the public, commercial or not-for-profit sectors.

Competing interest statement
All authors declare that they have no conflict of interest.

Ethics approval statement
The protocol was not required ethics approval, because it does not involve research on human participants
